# Serum β-synuclein, neurofilament light chain and glial fibrillary acidic protein as prognostic biomarkers in moderate-to-severe acute ischemic stroke

**DOI:** 10.1038/s41598-023-47765-7

**Published:** 2023-11-28

**Authors:** Lorenzo Barba, Christoph Vollmuth, Samir Abu-Rumeileh, Steffen Halbgebauer, Patrick Oeckl, Petra Steinacker, Alexander M. Kollikowski, Cara Schultz, Judith Wolf, Mirko Pham, Michael K. Schuhmann, Peter U. Heuschmann, Karl Georg Haeusler, Guido Stoll, Hermann Neugebauer, Markus Otto

**Affiliations:** 1 Department of Neurology, Martin-Luther-University of Halle-Wittenberg, Ernst-Grube Strasse 40, 06120 Halle (Saale), Germany; 2 Department of Neurology, University of Würzburg, Würzburg, Germany; 3 Department of Neurology, University of Ulm, Ulm, Germany; 4 German Center for Neurodegenerative Diseases (DZNE E.V.), Ulm, Germany; 5 Department of Neuroradiology, University of Würzburg, Würzburg, Germany; 6 Institute for Clinical Epidemiology and Biometry, University of Würzburg, Würzburg, Germany

**Keywords:** Diseases of the nervous system, Neuroimmunology

## Abstract

We aimed to assess the prognostic value of serum β-synuclein (β-syn), neurofilament light chain (NfL) and glial fibrillary acidic protein (GFAP) in patients with moderate-to-severe acute ischemic stroke. We measured β-syn, GFAP and NfL in serum samples collected one day after admission in 30 adult patients with moderate-to-severe ischemic stroke due to middle cerebral artery (MCA) occlusion. We tested the associations between biomarker levels and clinical and radiological scores (National Institute of Health Stroke Scale scores, NIHSS, and Alberta Stroke Program Early CT Score, ASPECTS), as well as measures of functional outcome (modified Rankin Scale, mRS). Serum biomarkers were significantly associated with ASPECTS values (β-syn p = 0.0011, GFAP p = 0.0002) but not with NIHSS scores at admission. Patients who received mechanical thrombectomy and intravenous thrombolysis showed lower β-syn (p = 0.029) und NfL concentrations (p = 0.0024) compared to patients who received only mechanical thrombectomy. According to median biomarker levels, patients with high β-syn, NfL or GFAP levels showed, after therapy, lower clinical improvement (i.e., lower 24-h NIHSS change), higher NIHSS scores during hospitalization and higher mRS scores at 3-month follow-up. Elevated serum concentrations of β-syn (p = 0.016), NfL (p = 0.020) or GFAP (p = 0.010) were significantly associated with 3-month mRS of 3–6 vs. 0–2 even after accounting for age, sex and renal function. In patients with moderate-to-severe acute ischemic stroke, serum β-syn, NfL and GFAP levels associated with clinical and radiological scores at different timepoints and were able to predict short- and middle-term clinical outcomes.

## Introduction

In recent years, blood-based biomarkers have been largely investigated to better prognosticate patients with acute ischemic stroke^1^. Among the most promising ones, neurofilament light chain protein (NfL) and glial fibrillary acidic protein (GFAP) represent surrogates of neuroaxonal and glial injury, respectively^2, 3^. In previous studies on minor-to-moderate ischemic stroke, NfL and GFAP showed a fair value as prognostic factors to predict short- and middle-term functional outcomes as well as all-cause mortality^2–4^. However, the role of such markers in patients with severe ischemic stroke has been only partly investigated^5^. In addition, β-synuclein (β-syn) is emerging as a biomarker for synaptic damage in several neurological disorders, such as neurodegenerative^6–9^ and traumatic diseases^10^. The promising results obtained so far encourage the assessment of blood β-syn also in cerebrovascular diseases, where its profile is, to date, still unexplored. These facts considered, the present exploratory analysis of a prospective single-center cohort aimed to test the possible associations of serum β-syn, NfL and GFAP levels with clinical and radiological scores and with measures of functional outcome in patients with moderate-to-severe acute ischemic stroke.

## Methods

### Inclusion criteria

We included patients with acute ischemic stroke due to middle cerebral artery (MCA) occlusion in the M1 segment, enrolled into a single-center perspective cohort (DRKS00022064) at the University of Würzburg (Germany) between 07/2020 and 08/2021 (detailed patients selection from the main cohort in Supplementary Fig. S1), which has been partly published elsewhere^11^. Inclusion criteria were a clear time window from clinical onset and an admission National Institute of Health Stroke Scale (NIHSS) score ≥ 6 and/or acute treatment with mechanical thrombectomy (MT). From all patients (n = 30), we collected baseline data on the Alberta Stroke Program CT Score (ASPECTS) and NIHSS scores. After treatment (described in detail in the “Results” section), we collected data on: ASPECTS after 24–72 h^12, 13^; NIHSS scores after 24, 48 and 72 h and at discharge; modified Rankin Scale (mRS) at discharge and at 3-month follow-up.

### Blood sampling and analysis

Blood samples were collected one day after admission at scheduled timepoints (10am) (median time from clinical onset: 22 h 40 m; interquartile range, IQR 18 h 45 m–23 h 04 m) and analyzed according to standard procedures. We measured NfL by using a commercial kit for the Ella microfluidic system (BioTechne, Minneapolis, USA), GFAP with a commercial Simoa kit on a HD-X platform (Quanterix Inc., Lexington, USA) and β-syn with our recently described in-house established digital immunoassay^14^. In all measurements, the intra- and inter-assay coefficients of variability were < 10% and < 15%, respectively.

### Statistical analysis

Statistical analysis was performed with GraphPad 8 (GraphPad Software, La Jolla, USA) and R v4.2.2 (R Foundation, Vienna, Austria). We used the χ^2^ test and the Mann–Whitney test for two-group comparisons of categorical and continuous variables, respectively. For comparisons among multiple groups, we adopted the Kruskal–Wallis test. We calculated correlations between biomarker levels with the Spearman’s coefficient. Linear regression models were used to test associations between baseline characteristics and biomarker levels, as well as between biomarker levels and continuous clinical outcomes. Logistic regression models were adopted to test associations of serum biomarkers with binary outcomes^12, 13, 15^ (see section “Definition of binary outcome measures”). Multivariable analyses were adjusted for age, sex and creatinine levels. Nagelkerke’s R^2^ was used to estimate the explained variance of regression models. To test the diagnostic accuracy of biomarkers, we performed receiver operating characteristic (ROC) analysis. Biomarker concentrations were log-transformed for all analyses and p-values < 0.05 were considered statistically significant.

### Definition of binary outcome measures

We explored the associations of serum biomarkers with the following binary outcomes: (i) postinterventional ASPECTS ≥ 8 (n = 14) vs. < 8 (n = 16), which was previously associated with the functional outcome at 3-month follow-up^13^; (ii) NIHSS change within 24 h (ΔNIHSS = baseline NIHSS-24 h NIHSS) ≥ 4 (n = 13) vs. < 4 (n = 17), given its association with the functional outcome at 3-month follow-up^15^; (iii) death due to neurological complications (n = 5) vs. all other patients (n = 25); (iv) mRS of 0–2 (n = 10) vs. 3–6 (n = 20) at 3-month follow-up.

### Study protocol approval

This study was conducted in accordance with the Helsinki Declaration and its recent modifications. All patients gave written informed consent to participate in the research and the local Ethics Committee of the University of Würzburg approved the study protocol (reference n. 05/20-am).

## Results

### Baseline characteristics of the study population

In our study cohort, patients had a mean age of 75.1 (± standard deviation: 11.3) years and 17 participants (56.7%) were female. At admission, patients had median ASPECTS values of 7 (interquartile range, IQR 7–8) and median NIHSS scores of 14 (IQR 10–17). Patients were admitted after (median and IQR) 3 h 11 m (2 h 18 m–4 h 07 m) and blood samples were collected after (median and IQR) 22 h 40 m (18 h 45 m–27 h 04 m) from clinical onset. The Trial of Org 10172 in Acute Stroke Treatment (TOAST) classification system^16^ was used to distinguish different stroke etiologies, which were large-artery atherosclerosis in 5 cases, cardioembolism in 15 cases, other determined etiology in 4 cases and undetermined etiology in 6 cases. Moreover, 5 patients had previous neurological disorders in their medical history (dementia n = 1, Parkinson’s disease n = 1, previous stroke n = 1, delirium n = 2). Further clinical and biochemical data are reported in Table 1.

**Table 1:** Clinical, radiological and biochemical data of the study population.

	Total (n = 30)	3-month mRS 0–2 (n = 10)	3-month mRS 3–6 (n = 20)	p-value
Females/males	17/13	5/5	12/8	0.602
Age (years)	75.1 (± 11.3)	75.5 (± 9.4)	75.0 (± 12.4)	0.974
Time from onset to blood sampling	22 h 40 m (18 h 45 m–23 h 04 m)	20 h 53 m (15 h 38 m–24 h 8 m)	23 h 30 m (19 h 30 m–27 h 30 m)	0.140
Previous neurological diseases* (yes/no)	5/25	2/8	3/17	0.729
ASPECTS at admission	7 (7–8)	9 (7.5–9)	7 (6–8)	0.015
NIHSS at admission	14.0 (10.0–17.0)	13.0 (8.8–16.3)	14.0 (10.3–17.0)	0.656
Thrombectomy (yes/no)	27/3	9/1	18/2	1.00
Intravenous thrombolysis (yes/no)	11/19	5/5	6/14	0.284
ASPECTS after 24–72 h	7 (5–8)	7.5 (7–8.75)	6.5 (5–7)	0.061
NIHSS score change within 24 h	3 (− 2.5 to 9)	− 1 (− 3.75 to 3)	9 (7.5–12.3)	0.0001
24 h NIHSS	10.0 (4.3–14.8)	1.5 (0.8–6.3)	12.5 (8.3–21.5)	< 0.0001
48 h NIHSS	9.0 (1.0–14.0)	0.5 (0.0–4.0)	11.0 (9.0–22.0)	< 0.0001
72 h NIHSS	6.0 (1.0–14.0)	1.0 (0.0–3.3)	12.0 (6.0–26.0)	< 0.0001
NIHSS at discharge	3.0 (1.0–7.8)	0.5 (0.0–2.5)	7.5 (2.5–13.3)	0.0005
mRS at discharge	4.0 (1.3–5.8)	1.0 (1.0–2.5)	5.0 (4.0–6.0)	< 0.0001
mRS after 90 days	4.0 (1.3–6.0)	1.0 (1.0–2.3)	5.5 (4.0–6.0)	< 0.0001
Creatinine (mg/dl)	0.855 (0.740–1.213)	0.915 (0.773–1.060)	0.815 (0.710–1.250)	0.860
β-syn (pg/ml)	20.1 (5.8–43.8)	4.8 (1.9–25.1)	32.6 (9.2–94.4)	0.0023
NfL (pg/ml)	50.4 (34.0–113.1)	34.4 (19.3–49.8)	76.6 (42.1–178.8)	0.0011
GFAP (ng/ml)	6.3 (1.0–20.5)	0.5 (0.2–7.1)	9.2 (5.3–21.8)	0.0045

Age is reported as mean ± standard deviation, other continuous variables as median (interquartile range).

*1 dementia, 1 Parkinson’s disease, 1 previous stroke, 2 delirium.

### Associations between serum biomarker levels and baseline characteristics

At day 1, serum β-syn (median: 20.1 pg/ml, IQR 5.8–43.8 pg/ml), NfL (median: 50.4 pg/ml, IQR 34.0–113.1 pg/ml) and GFAP (median: 6.3 ng/ml, IQR 1.0–20.5 ng/ml) concentrations were not significantly associated with patients’ age, sex, time from clinical onset to blood sampling, admission NIHSS scores, creatinine levels, stroke etiology or presence of neurological comorbidities.

**Figure 1:**
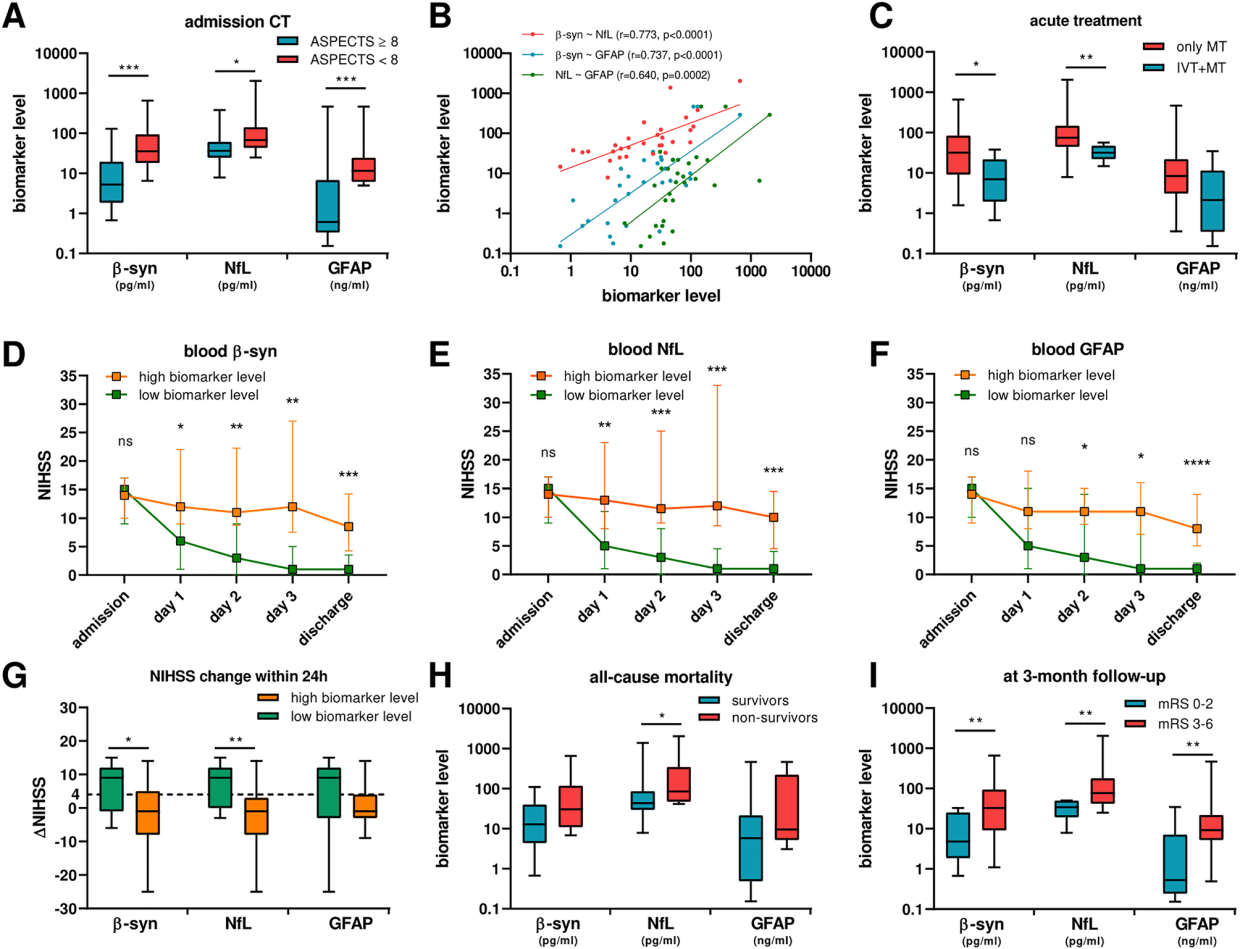
Serum biomarkers in patients with acute ischemic stroke. (**A**) Biomarker levels according to the admission ASPECTS values (≥ 8 n = 14 vs. < 8 n = 16); (**B**) Spearman’s correlations among biomarkers; (**C**) biomarker levels according to the treatment of choice (only MT n = 19 vs. IVT + MT n = 8); NIHSS progression according to median (**D**) β-syn (20.1 pg/ml), (**E**) NfL (50.4 pg/ml) and (**F**) GFAP (6.3 ng/ml) concentrations; (∆) 24-h NIHSS change (baseline NIHSS − 24-h NIHSS) according to median biomarker levels; (**H**) biomarker levels according to all-cause mortality (non-survivors n = 8 vs. survivors n = 22); (**I**) biomarker levels according to the 3-month mRS scores (0–2 n = 10 vs. 3–6 n = 20). *p < 0.05; **p < 0.01; ***p < 0.001.

Interestingly, we found significant associations between the ASPECTS values at admission and serum levels of β-syn [β: − 0.318 (95% confidence interval, 95%CI: − 0.489 to − 0.147), p = 0.0011] and GFAP [β: − 0.482 (95%CI: − 0.704to − 0.261), p = 0.0002], whereas the association with NfL did not reach statistical significance [β: − 0.149 (95%CI − 0.297 to − 0.0008), p = 0.059]. By distinguishing patients according to the admission ASPECTS, patients with ASPECTS < 8 (n = 16) had increased β-syn (p = 0.0006), NfL (p = 0.038) and GFAP (p = 0.0009) levels compared to patients with ASPECTS ≥ 8 (n = 14) (Fig. 1A). Finally, serum concentrations of β-syn, NfL and GFAP were well correlated one with each other at day 1, with strongest correlations observed between β-syn and NfL (r = 0.773, p < 0.0001, Fig. 1B).

### Associations between serum biomarkers and acute treatments

Treatment of choice was only mechanical thrombectomy (MT) in 19 cases, only intravenous thrombolysis (IVT) in 3 cases and both therapies (IVT + MT) in 8 cases. Patients who received only MT showed significantly higher serum concentrations of β-syn (p = 0.029) and NfL (p = 0.0024) compared to patients who received IVT + MT (Fig. 1C). Among patients who received MT (n = 27), the procedure was successful in 24 (88.9%) cases with modified Treatment In Cerebral Ischemia (mTICI) scores of 2b (n = 16, 59.3%), 2c (n = 5, 22.2%) or 3 (n = 3, 11.1%). The three remaining cases (11.1%) had mTICI scores of 0, 1 and 2a in one case each (3.7%). Patients with unsuccessful revascularization (i.e., mTICI 0-2a) showed increased levels of NfL (p = 0.046), but not of β-syn (p = 0.352) or GFAP (p = 0.914), compared to patients with favourable procedure (i.e., mTICI 2b-3) (Supplementary Fig. 2). Furthermore, the time to recanalization (median: 49 min, IQR 32–70 min) was not significantly associated with serum β-syn (p = 0.914), NfL (p = 0.314) or GFAP (p = 0.557) concentrations.

### Clinical and neuroradiological progression according to median biomarker levels

After stratification of patients according to median β-syn (20.1 pg/ml), NfL (50.4 pg/ml) and GFAP (6.3 ng/ml) concentrations, patients with higher biomarker levels (n = 15) had lower ASPECTS values and higher NIHSS scores after therapy than patients with low biomarker concentrations (n = 15), even though they did not differ in their admission NIHSS scores (Fig. 1D–F, Supplementary Table 1). Moreover, the former group experienced an overall lower clinical improvement soon after therapy (i.e., lower 24-h ΔNIHSS) compared to the latter group (Fig. 1G). Interestingly, we also observed significantly lower mRS scores in patients with high vs. low β-syn, NfL and GFAP levels both at discharge and at 3-month follow-up (Supplementary Table 1).

### Associations between biomarker levels and post-interventional clinical and radiological variables

Patients with post-interventional ASPECTS values < 8 (n = 21) showed higher serum levels of β-syn (p = 0.0014) and GFAP (p = 0.008) compared to patients with ASPECTS ≥ 8 (n = 9), whereas the increase of NfL was of borderline significance (p = 0.05) (Supplementary Fig. 2). At univariate logistic regression analysis, both β-syn and GFAP were significantly associated with an ASPECTS < 8 vs. ≥ 8, but statistical significance did not survive after adjustment for age, sex and creatinine levels (Supplementary Table 2).

Similarly, patients with 24-h ΔNIHSS < 4 (n = 17) showed higher levels of β-syn (p = 0.002) and NfL (p = 0.015), as well as a tendence towards increased GFAP levels (p = 0.053), compared to patients with 24-h ΔNIHSS ≥ 4 (n = 13). However, only β-syn was significantly associated even at multivariate analysis with the 24-h ΔNIHSS both as a continuous (p = 0.002) and as a binary measure (i.e., < 4 vs. ≥ 4, p = 0.016) (Supplementary Tables 2 and 3).

### Associations between serum biomarkers and clinical outcomes

During the hospitalization period, 5 patients died because of severe neurological complications (malignant MCA infarction n = 3, hemorrhagic infarction n = 2) and 3 patients died because of severe non-neurological complications (i.e., aspiration pneumonia). Two additional patients died because of severe aspiration pneumonia before 3-month follow-up. In our cohort, only NfL levels were higher in non-survivors (n = 8) vs. survivors (n = 22, p = 0.031) (Fig. 1H). After accounting for the cause of death, patients who died because of severe neurological complications (n = 5) had significantly higher levels of β-syn (p = 0.027) and NfL (p = 0.019) compared to all other patients (n = 25), but not compared only to patients who died because of aspiration pneumonia (n = 5) (Supplementary Fig. 2). Moreover, Both β-syn and NfL were significantly positively associated with the risk of death due to severe neurological complications at univariate but not at multivariate logistic regression analysis (Supplementary Table 2).

Finally, patients with 3-month mRS of 3–6 showed elevated serum β-syn (p = 0.0023), NfL (p = 0.0011) and GFAP (p = 0.0045) concentrations compared to patients with 3-month mRS of 0–2 (Fig. 1I). Similar results were found when considering the mRS scores at discharge (Supplementary Fig. 2). Of notice, all three serum biomarkers were significant predictors of unfavourable outcome at 3-month follow-up both at uni- and at multivariate analysis after accounting for age, sex and renal function (Supplementary Table 2).

## Discussion

In the present study, we reported on the prognostic value of blood β-syn, NfL and GFAP in patients with moderate-to-severe acute ischemic stroke. Our preliminary data showed that β-syn and GFAP concentrations measured at day 1 after reperfusion were significantly associated with admission and post-interventional ASPECTS, suggesting a direct relationship between the biomarker levels and the extent of the ischemic injury. Instead, we found no associations between NfL and ASPECTS values at admission, which is in line with previous studies that reported associations with infarct volume when the biomarker was measured after 3 to 7 days from the ischemic event but not within 24 h^4, 17^. Furthermore, the lack of significant associations between β-syn, NfL or GFAP concentrations and baseline NIHSS score could be due to the lack of a strict relationship between NIHSS scores and ASPECTS^18^. Alternatively, such associations might be detectable only in patients with minor stroke^17^.

On another issue, it is still poorly explored what impact different types of acute treatment (i.e., MT and IVT alone or in combination) may have on brain-specific biomarkers^5, 19, 20^. Our preliminary results suggest that the use of IVT in addition to mechanical revascularization may be associated with lower biomarker levels compared to patients who received only MT, but larger confirmative studies are needed. Most interestingly, our findings suggest a possible role of serum biomarkers for monitoring clinical progression at different timepoints after therapy, both during hospitalization and at follow-up. We could largely confirm previous results on the good prognostic value of blood NfL and GFAP in minor-to-moderate acute ischemic stroke^2–4^, but also highlight the potential utility of such biomarkers for severely affected patients^5^. Indeed, serum NfL seemed to be more associated with all-cause mortality, in line with previous evidence in healthy elderlies^21^. Instead, GFAP blood concentrations may change with a rapid dynamics within few hours after stroke and acute treatment^5, 10^, suggesting that its prognostic value might be strictly time-dependent. Hence, blood-based biomarkers with different temporal patterns might be used in combination for prognostication during distinct disease stages.

As a strength of our work, we included in the analysis factors that are well-known in literature for influencing serum biomarker levels, such as age and renal function^22^. On the other hand, the small sample size represents the major limitation of our exploratory single-center study and multiple testing in such sample might increase the risk of false-positive results. Moreover, blood collection was performed only at a single timepoint during the acute post-treatment phase. Other studies showed that the performance of some biomarkers (e.g., NfL) for predicting clinical outcomes in patients with ischemic stroke may be better when measured, for example, after 7 days from the event^3^. Instead, the temporal dynamics of GFAP and β-syn in cerebrovascular diseases, as well as their possible utility when measured at later timepoints, remains still unexplored. Moreover, we observed in our cohort a higher proportion of patients who died because of aspiration pneumonia compared to previous data from a recent meta-analysis^23^, which could be due to an older age at onset and a higher disease severity. To date, the prognostic value of blood biomarkers in relation to different causes of death has not been assessed yet. Hence, our preliminary results should be replicated in independent and larger cohorts to address these missing points.

## Conclusions

In conclusion, serum β-syn levels were associated with clinical and radiological scores of stroke severity and with measures of functional outcome in this exploratory analysis. Moreover, NfL and GFAP showed distinct prognostic utility. Further studies on larger cohorts should better test the possible additive prognostic role of β-syn if compared to NfL and GFAP.

### Supplementary Information

Supplementary Information 1.

Supplementary Information 2.

Supplementary Figure 1.

Supplementary Figure 2.

## Data Availability

Complete data of this study are available upon reasonable request from the corresponding author.

## Abbreviations

ASPECTS: Alberta Stroke Program CT Score
β-syn: β-Synuclein
GFAP: Glial fibrillary acidic protein
mRS: Modified Rankin Scale
NfL: Neurofilament light chain protein
NIHSS: National Institute of Health Stroke Scale
ΔNIHSS: NIHSS change (baseline NIHSS - 24-h NIHSS)
