# Supplementary material for: Serum β-synuclein, neurofilament light chain and glial fibrillary acidic protein as prognostic biomarkers in moderate-to-severe acute ischemic stroke
Source: Sci Rep. 2023 Nov 28;13:20941. doi: 10.1038/s41598-023-47765-7 (PMC10684607; doi:10.1038/s41598-023-47765-7)
Supplement: Supplementary file 1 — Supplementary Information 1. [file 41598_2023_47765_MOESM1_ESM.docx]

**Appendix.** Authors’ affiliations and contributions.

| Name | Affiliation | Contribution |
| --- | --- | --- |
| Lorenzo Barba, MD | Department of Neurology, Martin-Luther University of Halle-Wittenberg, Germany | Conception and design of the study, biomarker analysis, interpretation of results, statistical analysis, first draft of the manuscript |
| Christoph Vollmuth, MD | Department of Neurology, University of Würzburg, Würzburg, Germany | Conception and design of the study, study management and coordination, interpretation of results, statistical analysis, first draft of the manuscript |
| Samir Abu-Rumeileh, MD | Department of Neurology, Martin-Luther University of Halle-Wittenberg, Germany | Conception and design of the study, interpretation of results, statistical analysis, revised the manuscript for intellectual content |
| Steffen Halbgebauer, PhD | Department of Neurology, University of Ulm, Germany | Biomarker analysis, interpretation of results, statistical analysis, revised the manuscript for intellectual content |
| Patrick Oeckl, PhD | Department of Neurology, University of Ulm, Germany  German Center for Neurodegenerative Diseases (DZNE e.V.), Ulm, Germany | Biomarker analysis, interpretation of results, statistical analysis, revised the manuscript for intellectual content |
| Petra Steinacker, PhD | Department of Neurology, Martin-Luther University of Halle-Wittenberg, Germany | Biomarker analysis, interpretation of results, statistical analysis, revised the manuscript for intellectual content |
| Alexander M. Kollikowski, MD | Department of Neuroradiology, University of Würzburg, Würzburg, Germany | Collection of clinical and radiological data, interpretation of results, revised the manuscript for intellectual content |
| Cara Schultz | Department of Neurology, University of Würzburg, Würzburg, Germany | Collection of clinical and radiological data, interpretation of results, revised the manuscript for intellectual content |
| Judith Wolf | Department of Neurology, University of Würzburg, Würzburg, Germany | Collection of clinical and radiological data, interpretation of results, revised the manuscript for intellectual content |
| Mirko Pham, MD | Department of Neuroradiology, University of Würzburg, Würzburg, Germany | Collection of clinical and radiological data, interpretation of results, revised the manuscript for intellectual content |
| Michael K. Schumann, PhD | Department of Neurology, University of Würzburg, Würzburg, Germany | Collection of clinical and radiological data, interpretation of results, revised the manuscript for intellectual content |
| Peter U. Heuschmann, MD | Institute for Clinical Epidemiology and Biometry, University of Würzburg, Würzburg, Germany | Statistical analysis, interpretation of results, revised the manuscript for intellectual content |
| Karl Georg Haeusler, MD | Department of Neurology, University of Würzburg, Würzburg, Germany | Collection of clinical and radiological data, interpretation of results, revised the manuscript for intellectual content |
| Guido Stoll, MD | Department of Neurology, University of Würzburg, Würzburg, Germany | Collection of clinical and radiological data, interpretation of results, revised the manuscript for intellectual content |
| Hermann Neugebauer, MD | Department of Neurology, University of Würzburg, Würzburg, Germany | Conception and design of the study, study management and coordination, interpretation of results, statistical analysis, first draft of the manuscript |
| Markus Otto, MD | Department of Neurology, Martin-Luther University of Halle-Wittenberg, Germany | Conception and design of the study, study management and coordination, interpretation of results, statistical analysis, first draft of the manuscript |
