# Supplementary material for: Serum β-synuclein, neurofilament light chain and glial fibrillary acidic protein as prognostic biomarkers in moderate-to-severe acute ischemic stroke
Source: Sci Rep. 2023 Nov 28;13:20941. doi: 10.1038/s41598-023-47765-7 (PMC10684607; doi:10.1038/s41598-023-47765-7)
Supplement: Supplementary file 2 — Supplementary Information 2. [file 41598_2023_47765_MOESM2_ESM.docx]

**Serum β-synuclein, neurofilament light chain and glial fibrillary acidic protein as prognostic biomarkers in moderate-to-severe acute ischemic stroke**

Lorenzo Barba^1*^, MD; Christoph Vollmuth^2*^, MD; Samir Abu-Rumeileh^1^, MD; Steffen Halbgebauer^3^, PhD; Patrick Oeckl^3,4^, PhD; Petra Steinacker^1^, PhD; Alexander M. Kollikowski^5^, MD; Cara Schultz^2^, Judith Wolf^2^, Mirko Pham^5^, MD; Michael K. Schuhmann^2^, PhD; Peter U. Heuschmann^6^, MD; Karl Georg Haeusler^2^, MD; Guido Stoll^2^, MD; Hermann Neugebauer^2**^, MD; Markus Otto^1**^, MD

^1^Department of Neurology, Martin-Luther-University of Halle-Wittenberg, Halle (Saale), Germany

^2^Department of Neurology, University of Würzburg, Würzburg, Germany

^3^Department of Neurology, University of Ulm, Ulm, Germany

^4^German Center for Neurodegenerative Diseases (DZNE e.V.), Ulm, Germany

^5^Department of Neuroradiology, University of Würzburg, Würzburg, Germany

^6^Institute for Clinical Epidemiology and Biometry, University of Würzburg, Würzburg, Germany

*contributed equally

**contributed equally

**Supplementary Table 1.** Clinical, radiological and biochemical data according to median serum biomarker levels.

|  | **β-syn (cut-off: 20.1 pg/ml)** |  |  | **NfL (cut-off: 50.4 pg/ml)** |  |  | **GFAP (cut-off: 6.3 ng/ml)** |  |  |
| --- | --- | --- | --- | --- | --- | --- | --- | --- | --- |
|  | **low level** | **high level** | **p-value** | **low level** | **high level** | **p-value** | **low level** | **high level** | **p-value** |
| **Females/males** | 8/7 | 9/6 | 0.71 | 8/7 | 9/6 | 0.71 | 9/6 | 8/7 | 0.71 |
| **Age (years)** | 75.9 (±9.2) | 74.4 (±13.4) | 0.89 | 75.5 (±11.0) | 74.7 (±12.0) | 0.86 | 76.5 (±8.4) | 73.8 (±13.8) | 0.85 |
| **ASPECTS at admission** | 8 (7.5-9) | 7 (6-7) | 0.0036 | 8 (7.5-9) | 7 (6-7) | 0.0008 | 8 (7.5-9) | 7 (6-7) | 0.0036 |
| **NIHSS at admission** | 15 (9.5-17) | 14 (10.5-16.5) | 0.91 | 15 (9.5-16.5) | 14 (10.5-17) | 0.78 | 15 (10.5-17) | 14 (9.5-16.5) | 0.89 |
| **Thrombectomy (yes/no)** | 13/2 | 14/1 | 0.54 | 13/2 | 14/1 | 0.54 | 13/2 | 14/1 | 0.54 |
| **Thrombolysis (yes/no)** | 8/7 | 3/12 | 0.058 | 9/6 | 2/13 | 0.008 | 8/7 | 3/12 | 0.058 |
| **ASPECTS after 24-27h** | 8 (7-8.5) | 5 (2.5-6.5) | <0.0001 | 7 (7-8) | 6 (3.5-7) | 0.0063 | 7 (7-8) | 6 (5-7) | 0.054 |
| **NIHSS score change within 24h** | 9 (-0.5 - 11) | -1 (-5.5 - 4.5) | 0.033 | 9 (1.5-11) | -1 (-7 - 3) | 0.0043 | 9 (-0.5 - 11) | -1 (-2 - 3.5) | 0.12 |
| **24h NIHSS** | 6 (1.5-10) | 12 (10-21) | 0.014 | 5 (1-11) | 13 (8.5-22.5) | 0.0019 | 5 (1-13.5) | 11 (8.5-16) | 0.065 |
| **48h NIHSS** | 3 (0.5-7.5) | 11 (9.5-21) | 0.0074 | 3 (0-7) | 11.5 (9.5-22.8) | 0.0006 | 3 (0-10) | 11 (9-13.5) | 0.039 |
| **72h NIHSS** | 1 (0.3-4) | 12 (9-20) | 0.0015 | 1 (0.3-4) | 12 (9-32) | 0.0003 | 1 (0.3-4) | 11 (8-16) | 0.019 |
| **NIHSS at discharge** | 1 (0-2.5) | 8.5 (5-13.3) | 0.001 | 1 (0-4) | 10 (7-14) | 0.001 | 1 (0-1.5) | 8 (5-12.5) | <0.0001 |
| **mRS at discharge** | 3 (1-4) | 5 (3.5-6) | 0.026 | 1 (1-3) | 5 (4.5-6) | 0.0005 | 1 (1-5) | 5 (4-5.5) | 0.026 |
| **mRS after 90 days** | 3 (1-4) | 5 (3-6) | 0.75 | 2 (1-3.5) | 6 (4-6) | 0.0013 | 2 (1-5) | 4 (3-6) | 0.059 |
| **Creatinine (mg/dl)** | 0.940 (0.755 - 1.125) | 0.820 (0.710 - 1.325) | 0.77 | 0.940 (0.670 - 1.175) | 0.810 (0.775 - 1.270) | 0.852 | 0.810 (0.690 - 1.125) | 0.890 (0.775 - 1.270) | 0.533 |
| **β-syn (pg/ml)** | 5.5 (3.0-8.8) | 45.7 (32.2-98.3) | <0.0001 | 5.5 (3.0-8.8) | 45.7 (29.8-98.3) | <0.0001 | 5.5 (3.0-12.8) | 37.9 (25.7-98.3) | 0.0001 |
| **NfL (pg/ml)** | 35.4 (25.6-47.3) | 92.4 (55.3-198.5) | 0.001 | 33.6 (25.6-39.6) | 120 (68-219) | <0.0001 | 35.4 (25.6-50.0) | 92.4 (55.3-168.5) | 0.002 |
| **GFAP (ng/ml)** | 2.1 (0.5-5.5) | 17.8 (7.0-30.2) | 0.0003 | 0.6 (0.4-4.0) | 17.8 (7.0-23.7) | <0.0001 | 0.6 (0.4-4.0) | 21.4 (11.6-30.2) | <0.0001 |

Data on age are reported as mean ± standard deviation, whereas for other continuous variables as median (interquartile range).

**Supplementary Table 2.** Predictive value of log-transformed serum biomarkers.

| **Binary outcome** | **Model** | **Variables** | **Models with β-syn** | | | | **Models with NfL** | | | | **Models with GFAP** | | | |
| --- | --- | --- | --- | --- | --- | --- | --- | --- | --- | --- | --- | --- | --- | --- |
|  |  |  | OR (95% CI) | p-value | R^2^ | AUC (95% CI) | OR (95% CI) | p-value | R^2^ | AUC (95% CI) | OR (95% CI) | p-value | R^2^ | AUC (95% CI) |
| **Postinterventional ASPECTS (<8 vs ≥8)** | Univariable model | biomarker | 18.31 (3.03 - 290.47) | 0.009 | 0.485 | 0.857 (0.715 - 0.999) | 11.39 (1.25 - 315.13) | 0.078 | 0.212 | 0.730 (0.524 - 0.937) | 5.37 (1.70 - 24.75) | 0.012 | 0.374 | 0.804 (0.624 - 0.985) |
|  | Multivariable model | biomarker | 517.2 (8.20 - >1000) | 0.056 | 0.683 | 0.926 (0.829 - 1.00) | 161.28 (4.33 - >1000) | 0.026 | 0.478 | 0.878 (0.732 - 1.00) | 34.36 (3.05 - >1000) | 0.067 | 0.583 | 0.889 (0.758 - 1.00) |
|  |  | age | 1.11 (0.94 - 1.38) | 0.269 |  |  | 1.007 (0.91 - 1.12) | 0.888 |  |  | 1.08 (0.95 - 1.30) | 0.298 |  |  |
|  |  | female sex | 0.04 (0.0004 - 0.72) | 0.083 |  |  | 0.046 (0.0012 - 0.52) | 0.037 |  |  | 0.013 (0.00001 - 0.53) | 0.124 |  |  |
|  |  | creatinine | 0.029 (0.0001 - 0.75) | 0.092 |  |  | 0.14 (0.01 - 1.45) | 0.095 |  |  | 0.04 (0.00012 - 1.43) | 0.137 |  |  |
| **NIHSS score change within 24h (<4 vs ≥4)** | Univariable model | biomarker | 8.63 (2.03 - 62.11) | 0.011 | 0.372 | 0.824 (0.671 - 0.975) | 10.56 (1.46 - 197.24) | 0.054 | 0.239 | 0.760 (0.582 - 0.939) | 3.16 (1.24 - 10.66) | 0.032 | 0.246 | 0.710 (0.498 - 0.923) |
|  | Multivariable model | biomarker | 8.03 (1.82 - 60.12) | 0.0156 | 0.430 | 0.833 (0.680 - 0.986) | 9.13 (1.01 - 204.3) | 0.096 | 0.290 | 0.760 (0.580 - 0.940) | 4.29 (1.33 - 21.12) | 0.034 | 0.373 | 0.805 (0.647 - 0.964) |
|  |  | age | 0.95 (0.86 - 1.05) | 0.331 |  |  | 0.96 (0.87 - 1.05) | 0.352 |  |  | 0.94 (0.85 - 1.03) | 0.214 |  |  |
|  |  | female sex | 2.20 (0.30 - 19.41) | 0.446 |  |  | 1.31 (0.20 - 9.20) | 0.775 |  |  | 3.61 (0.50 - 36.52) | 0.228 |  |  |
|  |  | creatinine | 9.10 (0.38 - 734.4) | 0.267 |  |  | 5.66 (0.35 - 239.9) | 0.319 |  |  | 27.36 (0.78 - >1000) | 0.104 |  |  |
| **Death due to neurological complications (yes vs no)** | Univariable model | biomarker | 10.11 (1.56 - 191.84) | 0.047 | 0.320 | 0.816 (0.597 - 1.00) | 9.46 (1.50 - 122.47) | 0.033 | 0.297 | 0.832 (0.625 - 1.00) | 2.76 (0.92 - 10.95) | 0.093 | 0.175 | 0.360 (0.074 - 0.646) |
|  | Multivariable model | biomarker | 5.27 (0.79 - 103.0) | 0.155 | 0.402 | 0.792 (0.575 - 1.00) | 5.79 (0.68 - 122.1) | 0.156 | 0.389 | 0.816 (0.615 - 1.00) | 1.82 (0.55 - 8.70) | 0.37 | 0.316 | 0.704 (0.420 - 0.989) |
|  |  | age | 0.97 (0.84 - 1.09) | 0.561 |  |  | 0.99 (0.85 - 1.15) | 0.871 |  |  | 0.96 (0.84 - 1.08) | 0.483 |  |  |
|  |  | female sex | 1.58 (0.12 - 32.36) | 0.727 |  |  | 1.40 (0.10 - 28.55) | 0.8 |  |  | 2.40 (0.19 - 67.30) | 0.535 |  |  |
|  |  | creatinine | 6.42 (0.50 - >1000) | 0.311 |  |  | 5.58 (0.40 - 983.8) | 0.346 |  |  | 13.65 (0.66 - >1000) | 0.213 |  |  |
| **mRS at follow-up (3-6 vs 0-2)** | Univariable model | biomarker | 8.14 (1.88 - 60.89) | 0.0147 | 0.352 | 0.835 (0.678 - 0.992) | 379.6 (7.42 - >1000) | 0.022 | 0.491 | 0.855 (0.721 - 0.989) | 7.95 (2.24 - 46.60) | 0.006 | 0.476 | 0.815 (0.599 - 1.00) |
|  | Multivariable model | biomarker | 8.66 (1.86 - 71.76) | 0.016 | 0.387 | 0.835 (0.676 - 0.994) | 468.1 (7.64 - >1000) | 0.020 | 0.503 | 0.865 (0.735 - 0.995) | 17.28 (3.09 - 328.5) | 0.010 | 0.577 | 0.895 (0.781 - 1.00) |
|  |  | age | 0.98 (0.88 - 1.09) | 0.723 |  |  | 0.97 (0.86 - 1.07) | 0.524 |  |  | 0.96 (0.84 - 1.08) | 0.51 |  |  |
|  |  | female sex | 2.760 (0.37 - 30.63) | 0.355 |  |  | 1.01 (0.10 - 10.28) | 0.99 |  |  | 8.01 (0.62 - 238.2) | 0.15 |  |  |
|  |  | creatinine | 2.72 (0.15 - 165.8) | 0.595 |  |  | 1.74 (0.05 - 109.25) | 0.779 |  |  | 47.28 (0.56 - >1000) | 0.118 |  |  |

Data derive from logistic regression analyses with the binary outcome as the dependent variable and the biomarker level as the independent variable. AUC values are obtained by means of receiver operating characteristic analysis.

**Supplementary Table 3.** Linear regression analysis of the associations between log-transformed serum biomarker levels and 24-hour ΔNIHSS as a continuous measure.

| **Model** | **Variables** | **Estimate (95%CI)** | **p-value** |
| --- | --- | --- | --- |
| Univariable model | β-syn | -7.63 (-11.69 - -3.57) | 0.001 |
| Multivariable model | β-syn | -8.08 (-12.60 - -3.56) | 0.002 |
|  | age | 0.02 (-0.27 - 0.31) | 0.876 |
|  | female sex | -1.29 (-7.20 - 4.62) | 0.673 |
|  | creatinine | 1.45 (-5.35 - 8.24) | 0.673 |
| Univariable model | NfL | -6.997 (-12.96 - -1.03) | 0.029 |
| Multivariable model | NfL | -7.20 (-14.13 - -0.27) | 0.052 |
|  | age | 0.007 (-0.33 - 0.34) | 0.967 |
|  | female sex | 0.09 (-6.70 - 6.88) | 0.980 |
|  | creatinine | 0.64 (-7.26 - 8.54) | 0.875 |
| Univariable model | GFAP | -3.55 (-6.92 - -0.19) | 0.048 |
| Multivariable model | GFAP | -3.68 (-7.48 - 0.13) | 0.070 |
|  | age | 0.04 (-0.29 - 0.38) | 0.790 |
|  | female sex | -1.49 (-8.25 - 5.27) | 0.670 |
|  | creatinine | -0.03 (-7.82 - 7.67) | 0.994 |

**Supplementary Fig. 1. Study protocol.**

**Supplementary Fig. 2.** **Serum biomarkers in patients with acute ischemic stroke.**

A) Serum biomarkers according to the mTICI scores (0, 1, 2a n=3 vs. 2b, 2c, 3 n=27); B) post-interventional ASPECTS values (<8 n=21 vs. ≥8 n=9); C) mortality due to severe neurological complications (yes n=5 vs. no=25); D) mRS scores at discharge (3-6 n=21 vs. 0-2 n=9); E) cause of death at 3-month follow-up (severe neurological complications n=5 vs. aspiration pneumonia n=5). *p<0.05; **p<0.01; ***p<0.001.
