# Supplementary figures and images for: Serum β-synuclein, neurofilament light chain and glial fibrillary acidic protein as prognostic biomarkers in moderate-to-severe acute ischemic stroke
Source: Sci Rep. 2023 Nov 28;13:20941. doi: 10.1038/s41598-023-47765-7 (PMC10684607; doi:10.1038/s41598-023-47765-7)

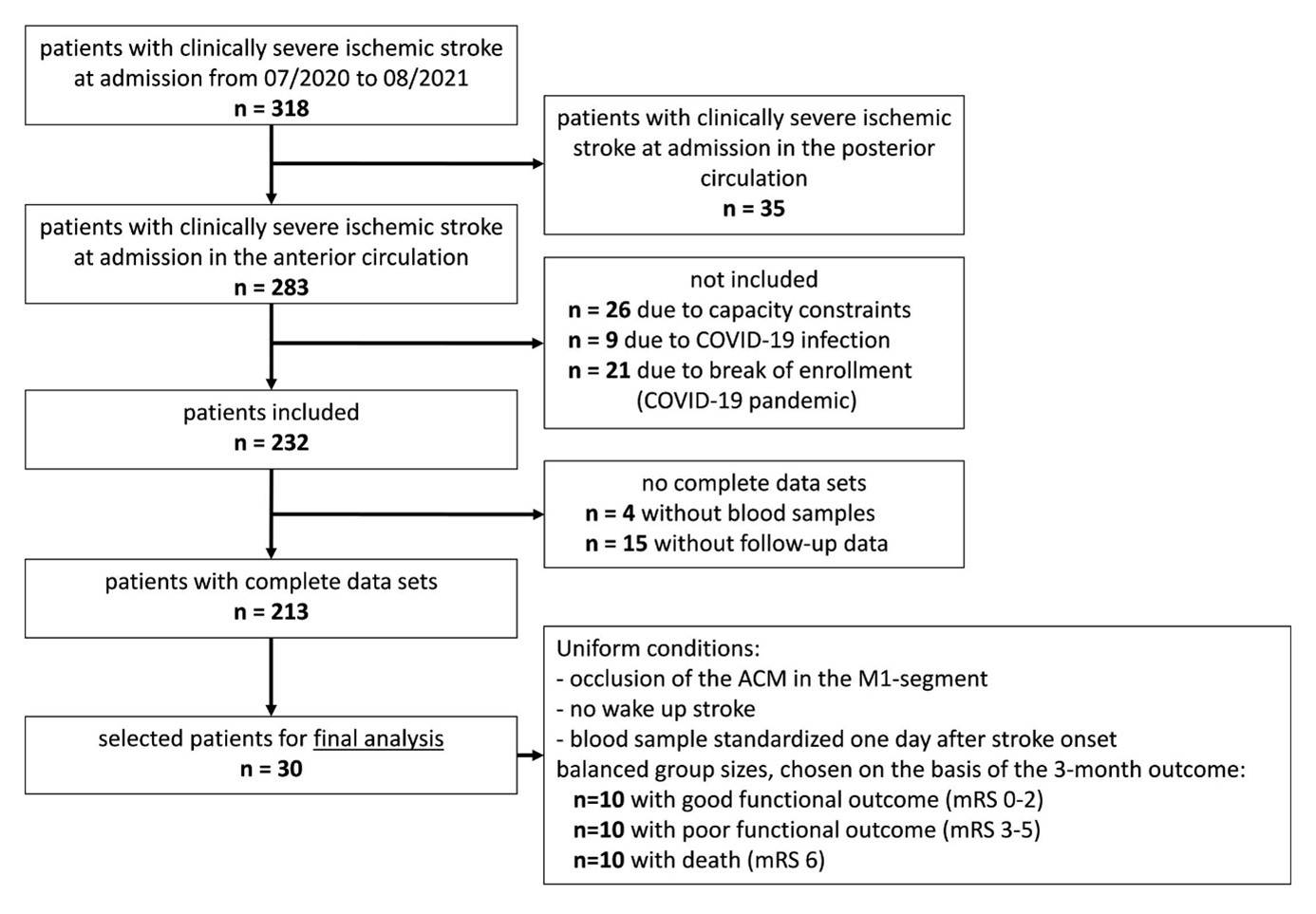

Supplement: Supplementary file 3 — Supplementary Figure 1. [file 41598_2023_47765_MOESM3_ESM.tiff]

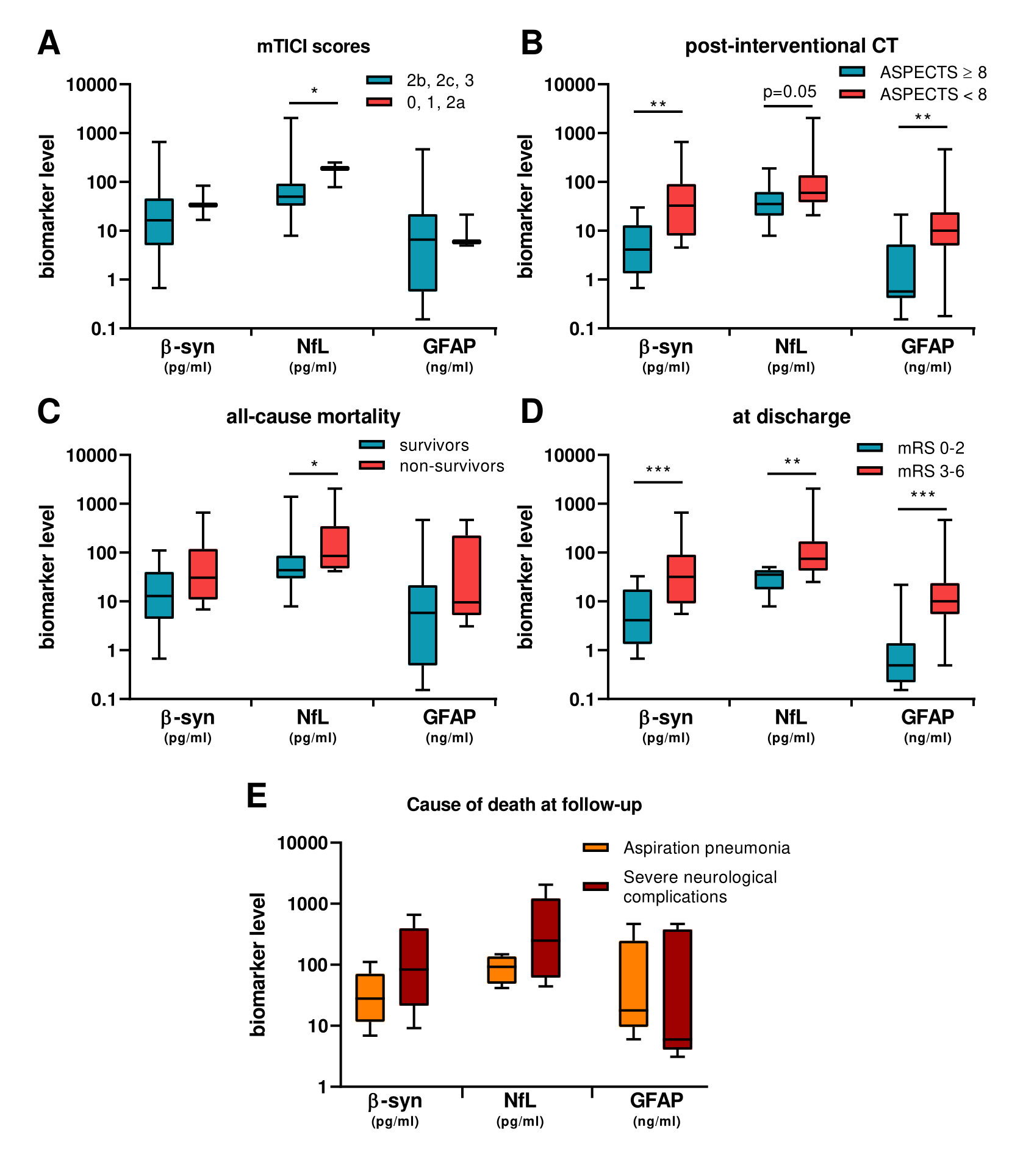

Supplement: Supplementary file 4 — Supplementary Figure 2. [file 41598_2023_47765_MOESM4_ESM.tiff]
